# On the Road to Precision Medicine: Magnetic Systems for Tissue Regeneration, Drug Delivery, Imaging, and Theranostics

**DOI:** 10.3390/pharmaceutics15071812

**Published:** 2023-06-24

**Authors:** Francesca Garello, Yulia Svenskaya, Bogdan Parakhonskiy, Miriam Filippi

**Affiliations:** 1 Molecular and Preclinical Imaging Centers, Department of Molecular Biotechnology and Health Sciences, University of Torino, Via Nizza 52, 10126 Torino, Italy; 2 Science Medical Center, Saratov State University, 410012 Saratov, Russia; yulia_svenskaya@mail.ru; 3 Faculty of Bioscience Engineering, Ghent University, Coupure Links 653, B-9000 Ghent, Belgium; bogdan.parakhonskiy@ugent.be; 4 Soft Robotics Laboratory, Department of Mechanical and Process Engineering, ETH Zurich, 8092 Zurich, Switzerland

Magnetic systems have always been considered as attractive due to their remarkable versatility. As proof of this, the number of articles concerning magnetic systems has dramatically increased over the past 20 years (Figure 1). In the pharmaceutical industry, magnetic systems are widely investigated both clinically and preclinically. Their strength lies in the ability to use them for imaging, therapy, cell stimulation, or guidance purposes. To target selected cells or pathological areas, magnetic systems can be functionalized with antibodies, peptides, or molecules that recognize specific molecular markers. In addition, these systems can be also loaded with drugs, thus resulting in platforms for simultaneous therapy and diagnosis (i.e., theranostics). This set of features makes magnetic systems attractive tools for personalized and precision medicine, in which therapy selection is tailored to a specific individual. As personalized medicine is expected to make modern medical methods more accessible, improve the control of personal health data, and drive the economic development of health technologies, magnetic systems will render future healthcare more equitable and efficient. Nevertheless, even if the impact of these magnetic tools on future personalized medical approaches is expected to be notable, this relation has not been extolled and carefully analyzed yet.

This Special Issue brings together magnetic systems and personalized medicine for the first time. Here, seven research articles and three comprehensive literature reviews were published. The first paper, authored by V.L. Kovalenko et al., reported on the synthesis and validation of multifunctional, biodegradable, and biocompatible magnetic nanoparticles with anti-cancer properties [1]. The nanoparticles were based on Poly Lactic-co-Glycolic Acid (PLGA), which was loaded with magnetite to quantitatively assess its accumulation in various organs and enable magnetic-assisted delivery. IR775 dye was then added to enable fluorescence in vivo imaging and Photodynamic Therapy (PDT), a promising strategy for the treatment of aggressive tumors. Finally, to selectively recognize the glycosylation profile of breast cancer cells, the nanoparticles were functionalized with lectin concanavalin A. Under external light irradiation, the resulting hybrid nanoparticles fully inhibited allograft solid tumor growth in murine models, thus demonstrating a great potential for combined magnetically assisted targeted delivery to tumor sites, tumor bioimaging, and treatment. Similarly, O. Y. Griaznova et al. reported on hybrid multimodal nanoparticles for the noninvasive imaging and therapy of cancer [2]. These researchers prepared bimetallic polyacrylic acid-coated Fe-Au core-satellite nanosystems to combine the magnetic and plasmonic properties of iron and gold nanoparticles in one core–shell. Then, they tested these systems as dual contrast agents for magnetic resonance imaging (MRI) and computed tomography (CT), and as sensitizers for the laser-induced hyperthermia of cancer cells. The particles were synthesized via pulsed laser ablation using the liquids (PLAL) method, which provided them with contaminated-free surfaces. While in the absence of photostimulation, the nanoparticles did not cause cytotoxicity on the cancer cell lines, and under laser irradiation, they induced a 100% cell death. When tested in vivo, the nanoparticles enabled the visualization of tumor boundaries in T_2_-weighted MR images and CT scans, thus proving the theranostic potential of this bimetallic system.

C. Iacoviță et al. investigated coating ferromagnetic iron oxide magnetic nanoparticles with silica layers of various thicknesses by adding different amounts of tetraethyl orthosilicate via a reverse microemulsion method [3]. The silica coating improved the nanosystems’ colloidal stability without affecting their magnetic properties and increased their biocompatibility and cellular uptake, enhancing their magnetic heating performance. A critical silica layer thickness was found, beyond which the nanoparticles’ colloidal stability decreased. The most stable formulation of coated nanoparticles presented an enhanced magnetic hyperthermia performance in water, and their specific absorption rate values increased by almost 1000 W/g_Fe_ compared to bare Fe_3_O_4_ nanoparticles. Finally, intracellular magnetic hyperthermia experiments revealed that the malignant cells were more sensitive to magnetic hyperthermia treatment compared to the normal ones. It was concluded that the controlled silica coating of ferromagnetic iron oxide nanoparticles enhanced their hyperthermia performance, cellular uptake, and destructive action on cancer cells, thus suggesting another design parameter that could be used to tune the performance of magnetic nanoparticles in biomedical applications. 

Three additional research articles reporting on magnetic systems designed for applications to specific pathological scenarios were collected. In more detail, the study by G. A. Soares et al. investigated how cirrhosis-associated hepatocarcinogenesis can alter the biodistribution of hepatic magnetic nanoparticles [4]. The researchers used a multichannel alternate current biosusceptometry system to real-time image the biodistribution of magnetic nanoparticles in the blood circulation and liver. Another customized sensor was used for ex vivo quantification of the MNPs that accumulated in the various organs. The investigated particles were manganese ferrite nanoparticles coated with citrate, which showed pharmacokinetic profiles that were remarkably affected by the pathophysiological factors induced by a cirrhosis state. Since the number of liver monocytes and macrophages did not vary, an altered hepatic blood flow likely caused an abnormal biodistribution and accumulation of nanoparticles injected into the cirrhotic animals. This study draws attention to the host pathophysiological state as a crucial parameter to be considered when designing in vivo nanosystems for optimized interactions between therapeutic agents and the injured target tissue. G.L. Lu et al. instead focused on using iron oxide nanoparticles for pain management [5]. A form of amine-terminated ultrasmall superparamagnetic iron oxide was found to have analgesic effects, even in the absence of any conjugated pain-relieving drug. Nevertheless, the cytotoxic risks of this nanoformulation were unclear. G.L. Lu and coworkers studied the effect of the nanoparticles’ toxicity on hippocampal long-term potentiation, revealing a double-side action of the nanosystems. These particles could relieve inflammatory pain in the spinal cord but also induce neurotoxicity in the central brain. The localized administration routes (e.g., intrathecal or intraplantar administration) of the nanoformulation did not elicit a toxicity response during the experiments performed by the authors; however, if the particles could leak to the brain and accumulate at high concentrations, they would have impaired hippocampal long-term potentiation. Finally, S.I. Bernad et al. focused on guiding magnetic nanoparticles through a stented artery model [6]. By applying external magnetic fields with a precisely positioned permanent magnet, polyethylene glycol-coated magnetic nanoparticles were remotely driven to a stented artery region, in which they aggregated and formed chains within and around the implanted stent. The performance of these magnetized, controllable nanoclusters as drug-loaded vectors for stented arteries was assessed. This contribution provided a new tool for site-specific drug delivery approaches based on the uniform-field-induced magnetization effect. 

Moving to paramagnetic systems, I. Pashkunova-Martic et al. designed two salinomycin (Sal)-based theranostic paramagnetic probes, comprising either gadolinium(III) or manganese(II) ions [7]. Salinomycin is a natural polyether antibiotic and a highly selective cytotoxic agent that forms complexes with metal(II) ions such as Zn^2+^, Cu^2+^, Co^2+^, and Ni^2+^. In this work, Sal was conjugated to Gd(III) or Mn(II) to obtain an MRI contrast agent and, to overcome the water insolubility of the two Sal complexes, they loaded them into empty bacterial ghost cells acting as transport vectors. The relaxivity values of the resulting systems were similar to those of the clinical MRI contrast agents, thus indicating that these probes can act as performant bioimaging agents. Considering such a contrast enhancement ability combined with strong cytotoxic activity, cancer theranostics could be the ideal applicative arena for these bio-hybrid nanotools.

In addition to original primary research, this Special Issue includes three comprehensive review articles that discuss the use of magnetic systems for personalized medicine from different points of view. S.H. Bossmann et al. described the current trends in applying iron-incorporated nanosystems to various areas of diagnostic imaging and drug delivery, focusing on cancer treatment, diagnosis, and wound care [8]. M. Cerqueira et al. provided an extensive overview of cancer theranostics with magnetic solid lipid nanoparticles being used in clinical trials [9], thus depicting the emergence of next-gen theranostic nanomedicines with potential for the selective, controlled, and safe delivery of chemotherapy. F. Garello et al. discussed micro and nanosystems for a magnetically targeted delivery of bioagents by describing the different classes of magnetic carriers that can serve as drug delivery platforms and their use in the magnetic guidance and delivery of bio-active agents (e.g., genes, drugs, and cells, etc.) [10]. Moreover, the authors highlighted the emergent applications of magnetic nano/microsystems of a synthetic or bio-hybrid composition (i.e., labeled cells) in regenerative medicine and tissue engineering, describing the magnetized bioagents that can be localized to specific target tissue following systemic or local administration and, remotely, noninvasively stimulated in vivo to promote tissue regenerative processes. Finally, the review presented the latest advances in combining magnetic targeting and imaging technologies to assist with drug delivery.

With the study collection presented in this Special Issue, we hope to guide scientists, clinicians, and other interested parties in designing magnetic systems for precision medicine and theranostics. Additional research based on creative, multidisciplinary collaboration will hopefully accelerate the progress in precision theranostics, aiming to use controllable magnetic nanotools for a better understanding and the personalized treatment of diseases. Hoping that readers will enjoy this Special Issue, we expect that our research content selection will catalyze debate and shape the future development of precision nanomedicine.

## Figures and Tables

**Figure 1:**
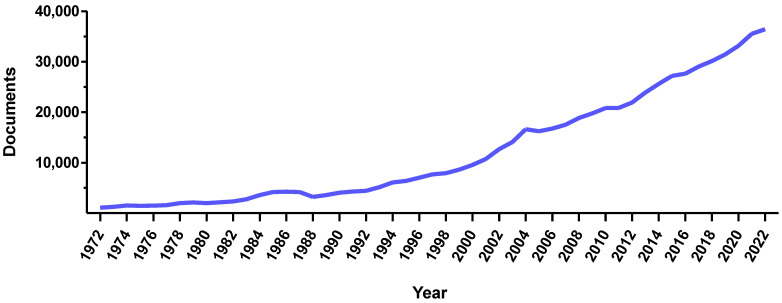
Increasing interest in magnetic systems. Number of documents published in the last 50 years that contain “magnetic systems” as a keyword (Source SCOPUS, accessed on 29 May 2023).

## Data Availability

Not applicable.
